# Polymer-Solvent Interactions in Modified Starches Pastes–Electrokinetic, Dynamic Light Scattering, Rheological and Low Field Nuclear Magnetic Resonance Approach

**DOI:** 10.3390/polym14152977

**Published:** 2022-07-22

**Authors:** Agnieszka Makowska, Krzysztof Dwiecki, Piotr Kubiak, Hanna Maria Baranowska, Grażyna Lewandowicz

**Affiliations:** 1 Department of Food Technology of Plant Origin, Poznań University of Life Sciences, 60-624 Poznań, Poland; agnieszka.makowska@up.poznan.pl; 2 Department of Biochemistry and Food Analysis, Poznań University of Life Sciences, 60-623 Poznań, Poland; krzysztof.dwiecki@up.poznan.pl; 3 Department of Biotechnology and Food Microbiology, Poznań University of Life Sciences, 60-627 Poznań, Poland; piotr.kubiak@up.poznan.pl; 4 Department of Physics and Biophysics, Poznań University of Life Sciences, 60-637 Poznań, Poland; hanna.baranowska@up.poznan.pl

**Keywords:** starch, ζ potential, hydrodynamic diameter, consistency index, flow behaviour index, spin-lattice relaxation time, spin-spin relaxation time, mean correlation time

## Abstract

Starch paste is a very complex dispersion that cannot be clearly classified as a solution, colloid or suspension and many factors affects its properties. As these ambiguities constitute a barrier to technological development, the aim of this study was to investigate the interaction of starch macromolecules with water by analysing the results of rheological properties, low field nuclear magnetic resonance (LF NMR), dynamic light scattering (DLS) and ζ potential analyses. Starch pastes with a concentration of 1%, prepared with distilled water and buffered to pH values of 2.5, 7.0 and 9.5 were analysed. It was proved that the pH buffering substantially decreased the values of consistency index but the pH value itself was not significant. LF NMR studies indicated that the dissolution of starch in water resulted in a reduction in spin-lattice as well as spin-spin relaxation times. Moreover, changes in relaxation times followed the patterns observed in rheological studies. Electrokinetic and DLS analyses showed that potential values are primarily influenced by the properties of the starches themselves and, to a lesser extent, by the environmental conditions. The conducted research also showed complementarity and, to some extent, substitutability of the applied research methods as well as exclusion chromatography (a method not used in this work).

## 1. Introduction

Starch, poly-α-glucan, is a polymer whose importance for food technology and human nutrition cannot be overestimated. The main plant crops, including corn, rice, wheat, potato and cassava, are rich sources of starch which is cheap and has become the main source of energy in human diet [1,2]. The enormous technological importance of starch is related to its ability to affect the texture of food products and, consequentially, improve their quality and shelf life. It is used in the production of dry mixtures for preparing puddings, jellies and fruit desserts. It is a popular thickening agent for different types of sauces, mainly ketchups, as well as a stabiliser of food emulsions such as mayonnaises and margarines. Starch is also a suitable ingredient for cured meat products where it is used to prevent leakage of water cause by heating during processing [3]. It is used in a wide variety of applications and fulfils multidirectional technological functions not only in food production [4]. Such a range of applications results not only from the differences in physicochemical properties of starch related to its biodiversity but also from the unusual susceptibility of this polysaccharide to modifications [5,6]. The latest trends in starch processing go beyond its traditional role of a hydrocolloid, even into the field of 3D printing [7,8]. Evaluation of the suitability of starch for individual applications is usually performed using rheological methods. Various instruments with different sensitivity, accuracy and reliability can be used for these types of analyses. Starch pastes are non-Newtonian fluids, rheometers are especially accurate in studying their rheological properties allowing analyses with a defined shear rate. On the other hand, the applicative importance of starch pasting characteristics for its technological suitability makes viscographs particularly useful [9,10]. New and improved analytical methods, which in the case of starch processing are mainly related to rheology, are developed. This development is stimulated by food technology and market demand for novel and competitive food products [11,12,13].

Rheological properties of starch are influenced by a number of factors. First of all, they are determined by the molecular mass of this polymer according to the Mark-Houwink equation. Other structural factors such as the number and length of branches are also of significant importance [14]. Moreover, chemical and physical modification also affect the viscosity of starch pastes [5,6,15]. The effect of substituents incorporated into the structure of starch macromolecules on the rheological properties of this biopolymer is related to the changes in polymer-solvent interactions. Polar substituents promote the hydration of starch macromolecules that results in a looser conformation and, as a consequence, higher viscosity. An inverse effect is caused by non-polar substituents. Degradation phenomena that accompany oxidation of starch result in a decrease in molecular mass and a decrease in viscosity. At lower degrees of modification, cross-linking which increases the molar mass of starch results in increased viscosity of pastes. Higher degrees of cross-linking impede starch dissolution and, paradoxically, results in reduced viscosity [16]. Changes in polymer-solvent interactions in starch pastes are also caused by the presence of different solutes. This creates an enormous technological challenge as starch in food interacts with complex matrices. Thus, the rheological properties of various binary and ternary systems containing starch have been extensively studied [17,18,19,20]. The effects of low-molecular-weight compounds commonly used in food production, i.e., salt and sugar, as well as some bioactive food ingredients, such as polyphenols, were studied [21,22,23,24]. Much attention has also been paid to the interaction of starch with high molecular mass components of food, such as polysaccharides and proteins [25,26,27,28,29,30,31,32]. In spite of the abundance of experimental data, it has not been possible so far to build a coherent theory that enables prediction of the rheological behaviour of starch in various environments. Such an attempt was made by application of cluster analysis grouping. However, it was only a fairly accurate summary of the effects of various low molecular mass compounds which did not take into consideration the status of starch macromolecules in the dispersion [33].

Low field nuclear magnetic resonance is a technique that makes it possible to study the effect of solutes present in the starch pastes. The physical basis of nuclear magnetic resonance (NMR) was given in the first half of the 20th century and initially it was used for studies in the field of physics. Due to the development of this technique, it soon became the basic tool for studying the structure of organic compounds. Currently the most important techniques of nuclear magnetic resonance include high resolution NMR (both in liquid and solid states), low field NMR and magnetic resonance imaging (MRI) [34]. The last one is particularly useful in medical applications. As NMR is particularly well-suited for the study of low molecular mass organic compounds, it is applied in the analyses of vitamins, antioxidants and other biologically active food components. In the case of NMR studies of larger molecules, such as polysaccharides or proteins, several challenges arise. ^1^H LF NMR analysis is based of proton relaxation phenomena, so it is designed for studying systems rich in water or fats. Food products contain water as an integral component, thus NMR relaxometry opens new possibilities to study the properties of these enormously complex mixtures [34,35,36,37]. LF NMR is an emerging methodology in food analysis. It was applied for studying fats, gels, emulsions as well as tissue-structured products [37,38,39,40,41,42,43,44].

Dynamic light scattering and ζ potential measurements have gained enormous popularity in recent years due to the development of nanotechnology and the need for comprehensive characterisation of nanoparticles, especially those of therapeutic relevance. Currently, table-top instruments are available which allow people to perform both the analyses in ordinary laboratory environments [45,46]. However, in the beginning, the development of these techniques, related to physical chemistry of colloids, was based on different physical phenomena. ζ potential measurements, based on electrokinetic phenomena, allows for the determination of the surface charge of particles dispersed in colloid systems. It is possible due to existence of surface charge that develops when two phases are placed in contact. Factors that influence ζ potential measurements include primarily pH and ionic strength of the dispersion. Its concentration should also be taken into consideration [45,46]. Dynamic light scattering, known also as photon correlation spectroscopy or quasi-elastic light scattering, is used for measuring the dimensions of particles (more precisely hydrodynamic radius and diffusion coefficient) dispersed in a liquid. Typically, it is conducted in the sub-micron region; however, the development of this technique enabled the measurement of the size of macromolecules in solution [45]. Light scattering phenomena are also employed for the determination of molecular mass o polymers (including starch). For this purpose, however, static light scattering signal, proportional to molar mass, the concentration of polymer and refractive index increment (dn/dc) of the solution, are recorded. It should be also mentioned that obtaining reliable results of these analyses demands achieving molecular dispersion of macromolecules in solution [14,47]. Factors that influence DLS results include: sample preparation conditions and its concentration, colour and fluorescence of samples, shape, agglomeration and rotational diffusion of nanoparticles.

Study on the ζ potential of potato and wheat starches proved that this parameter is pH dependent and is related to the surface properties of granules. Effective removal of ionic substances from the surface of granules of potato starch results in a stable value of ζ potential of about −11 mV [48]. The change in ζ potential can also be obtained by changing the size of starch particles. Subjecting native corn starch granules to high pressure homogenisation reduces their dimensions from micro- to nano-metre which in turn results in change in ζ potential from −43 to −62 mV [49]. In the case of corn starch nanocrystals obtained by acid hydrolysis, both ζ potential and size were found to be pH dependent. Due to the presence of carboxyl and sulphate groups on the surface of starch crystals, at acidic pH they reveal ζ potential of −7 mV and form agglomerates. An increase in pH causes the agglomerates to break. In alkaline pH, nanocrystals reveal ζ potential of −35 mV and form a transparent nanodispersion [50]. Similar results were obtained in studies on waxy maize starch. Moreover, it was shown that for pH values lower than 9, the average size of the particles was slightly affected by pH, whereas the ionic strength exhibited a more pronounced effect [51]. Incorporation of oxidised groups into waxy corn starch by hypochlorite treatment also results in the increase in absolute value of ζ potential [52]. Other methods of chemical modification, especially esterification and acid hydrolysis, were proposed for increasing the absolute value of the ζ potential and improving the stability of nanodispersions [53,54]. The influence of the presence of chemical modifying groups is so significant that it allows for the determination of the degree of substitution of starch phosphates by ζ potential measurement [55,56]. Electrostatic interactions between macromolecules, that can be expressed by ζ potential, strongly affect the pasting properties of starch. Studies on the effect of xanthan gum on the pasting characteristics of native potato starch as well as its cationic and anionic derivatives have shown that the ζ potential values of the resulting pastes are not additive. The absolute value of ζ potential of mixtures of xanthan and potato starch was higher than what could be expected from simple summation of the values of pure xanthan and starch. These data suggest a huge applicative relevance of ζ potential for the studies of the status of starch macromolecules in solution [57]. DLS and ζ potential studies are also useful in the studies of the effect of surfactants on the properties of corn starch-glycerol based dispersions and films. Whereas the presence of sorbitan monopalmitate, monostearate or monooleate has little effect on the particle size, the influence on ζ potential values in dispersion is significant. At the same time, the presence of surfactants significantly affects oxygen and water vapour permeability as well as tensile strength, elastic modulus, and elongation of films [58]. These findings suggest the usefulness of DLS and ζ potential studies in the development of bioplastic technology, especially with regard to thermoplastic starch films with improved water resistance and barrier properties. Studies of the ζ potential have also proved fruitful in determining the suitability of modified starches for use as stabilizers in the production of yoghurt. At pH = 4.0, typical for yoghurt, casein reveals positive ζ potential (21.4 mV), whereas native potato starch and modified starches show negative values negative (in the range −3.4–−15.5 mV). In protein–starch mixtures, due to attraction forces, zeta potential alters to values near zero [59].

Starch, both in its native and pasted form, is a very complex dispersion that cannot be clearly classified as a solution, colloid or suspension. Moreover, the properties of starch pastes are affected by many factors, primarily pH and the presence of different solutes. The selection of appropriate starch for a specific application is still a challenge for technologists. Therefore, the aim of this study was to provide new information in order to facilitate the understanding of interactions of starch macromolecules with water. To this end, results of rheological, LF NMR, DLS and ζ potential analyzes were studied.

## 2. Materials and Methods

### 2.1. Materials

Native potato starch and food grade modified starches–oxidised starch (E 1404), distarch phosphate (E 1412), acetylated starch (E 1420), were kindly provided by WPPZ SA (Luboń, Poland). Laboratory manufactured starch sodium octenyl succinate (E 1450) and cationic starch were also tested. Starch sodium octenylsuccinate (OSA starch) was synthesised in a water suspension with octenylsuccinic anhydride as the esterifying agent (SPI Supplies, West Chester, PA, USA), according to the procedure described by Jeon et al. [60]. Cationic starch was obtained in a water suspension with 3-chloro-2-hydroxy-N,N,N-trimethyl propyl ammonium chloride 99% (Sigma-Aldrich Co., St. Louis, MO, USA) as the etherifying agent.

### 2.2. Methods

Degrees of substitution of food grade modified starches were determined according to JECFA recommendations [61]. For cationic starches, the degree of substitution was determined based on nitrogen content according to EN ISO 3188 standard [62].

#### 2.2.1. Preparation of Samples for Analyses

Samples for rheological, LF NMR, DLS, and ζ potential analyses were prepared in a two-step manner. Firstly, gelatinisation of 2% starch suspensions was performed, by continuous stirring, for 30 min, followed by sterilisation at a temperature of 121 °C for 15 min. Secondly, after cooling, phosphate buffer was introduced in order to obtain pastes with known pH values and the starch concentration in the pastes was adjusted to 1%.

#### 2.2.2. Rheological Properties

Rheological properties were determined with RheoStress1 rheometer (Haake Technik GmbH, Vreden, Germany) equipped with a DG43 sensor. Tests were performed in CS mode. Shear stress was ramped from 0 Pa at time 0 s to 1.5 Pa at 120 s. Samples were thermostated at 25 °C. RheoWin 3 software (Haake Technik GmbH, Vreden, Germany) was used to determine the parameters of the Ostwald-de Waele model.

#### 2.2.3. LF NMR Analyses

Samples for LF NMR analyses were put into NMR tube (1.5 mL). The vessels were sealed with Parafilm^®^. The relaxation times of spin-lattice *T*_1_ and spin-spin *T*_2_, were measured using a pulse spectrometer PS15T (Ellab, Poznań, Poland). The device operates at the frequency of 15 MHz and includes an integrated system of temperature control. In order to measure of spin-lattice relaxation times, inversion-recovery pulse sequence was used [63]. Distance between impulses (*T*_1_) was changed from 100 to 1300 ms. Repetition time TR was 20 s, and a single sequence consisted of 32 repetitions. 119 measurement points from each of the 32 FID signals were collected in order to carry out calculation. Measurements of spin-spin relaxation times were carried out with CMPG impulses [63]. Distance between impulses was 8 ms. Measurements of both relaxation times were carried out in the 20 °C. Mean correlation time *τ_c_*, corresponding to the time necessary for one radian rotation of molecule, is derived from directly measured spin-lattice *T*_1_ and spin-spin *T*_2_ relaxation times [64]:

(1) $$\frac{1}{T_{1}}=2A\left(\frac{\tau_{c}}{1+{\left(\varpi \tau_{c}\right)}^{2}}+\frac{4\tau_{c}}{1+{\left(2\varpi \tau_{c}\right)}^{2}}\right)$$

(2) $$\frac{1}{T_{2}}=A\left(3\tau_{c}+\frac{5\tau_{c}}{1+{\left(\varpi \tau_{c}\right)}^{2}}+\frac{2\tau_{c}}{1+{\left(2\varpi \tau_{c}\right)}^{2}}\right)$$

where: *A* is a parameter characterizing the interacting spins, *ω* is a Larmor frequency.

#### 2.2.4. DLS and ζ Potential Analyses

Particle size (hydrodynamic diameter) in starch samples was determined using dynamic light scattering method. ζ potential was measured by electrophoretic light scattering. Zetasizer Nano ZS-90 (Malvern Pananalytical, Malvern, UK) was used in both measurements. ζ potential values are arithmetic mean of 6 independent measurements. Particle size was presented as z-average and the polydispersity index (PdI) was calculated (at least 3 independent measurements.

## 3. Results

### 3.1. Chemical Characteristics and Rheological Properties of Starch

Native potato starch and modified starches produced by introducing substituents of various chemical nature were studied. The modified starches were: oxidised starch (E 1404) of DS = 0.02, acetylated starch (E 1420) of DS = 0.02, starch sodium octenylsuccinate of DS = 0.02, distarch phosphate, and cationic starch of DS = 0.04. All investigated starches, with the exception of cationic starch, are popular thickeners and texture forming agents used in the food industry. Cationic starch is a wet end additive used in the paper industry to improve paper web strength and quality. The values of the degree of substitution of the studied starches differ from each other, nevertheless they are representative for the particular classes of modifications. The degree of substitution of distarch phosphate was not determined because the phosphorus content in modified potato starch samples does not reflect the degree of cross-linking (dissimilar to corn or wheat starch) [65]. This is due to the unique molecular structure of potato starch. While cereal starches contain phosphorus mainly in the form of phospholipids, potato starch contains it primarily in the form of phosphate monoesters bound to amylopectin [1,6,66]. Strong alkaline conditions necessary for cross-linking with sodium trimetaphosphate cause hydrolysis of the monoester phosphate bonds [65].

The choice of modified starches for the study was not accidental. Modifying groups incorporated into the structure of starch macromolecules cause multidirectional changes in the structure and physicochemical properties of this polysaccharide. Oxidised starch is manufactured by hypochlorite treatment. As a result, carboxyl groups are introduced into the structure of the hydrophilic; slightly anionic biopolymer giving it a stronger anionic character. Moreover, partial depolymerisation of amylose and amylopectin occurs, thus resulting in decreased viscosity of starch pastes [66]. Cross-linking with ester phosphate bonds also gives starch macromolecules a stronger anionic character. However, this process is accompanied by an increase in molecular mass and viscosity [5,6]. Acetylated starches contain relatively hydrophobic acetyl groups. As a result a decrease in pasting temperature and an increase in shear resistance with almost no change in viscosity are observed [6,67]. At the same time, the presence of acetyl groups results in enormous changes in starch-water interactions [16]. Sodium starch octenyl succinate contains long hydrophobic octenyl moiety and therefore is sometimes called hydrophobic starch [68]. However, it contains ionic carboxyl groups which gives it unique emulsifying properties and should rather be called amphiphilic starch. Cationic starch, due to the presence of quaternary ammonium groups, is the only one with cationic groups in the structure of macromolecules; it is this property that makes it so useful for increasing the strength of the paper web.

Analyses of the rheological properties were carried out for pastes with rather low starch concentration of 1%. Despite the low viscosity of analysed samples, it was possible to observe the effect of both–the type of chemical modification and the properties of solvent (Table 1). In general, the highest values of consistency index were recorded for pastes prepared with deionised water. The presence of buffer controlling pH substantially decreased values of consistency indexes. However, the pH value itself was not significant. In the case of native potato starch, only the paste prepared in deionised water revealed pseudoplastic behaviour. Flow behaviour index values above 0.8 indicated almost a Newtonian-like flow of all pastes prepared in buffers. The above observations are consistent with the literature data [17,22,23,33]. Newtonian-like flow is shown in low viscous pastes i.e., pastes characterised by low values of flow consistency indexes. This applies to virtually all pastes prepared in buffers, but also for acetylated, cationic and especially oxidized starch pastes in deionised water. Very low viscosity of oxidised starch pastes has also been reported in the literature as related to the partial degradation of starch macromolecules during processing with sodium hypochlorite [66]. Distarch phosphate pastes were characterised by the highest viscosity, which is related to the very high molar mass of the cross-linked starch macromolecules [5,6]. Comparison of the rheological behaviour of acetylated and OSA starches was found to be noteworthy. While acetylated starch revealed a more Newtonian-like flow than native potato starch, OSA starch manifested the contrary. Moreover, OSA starch seems to be more resistant to the effect of salts on rheological behaviour than all other starches. It should be emphasised that both modifications do not affect molecular mass of starch [60,69]. Cationic starch, in spite its unique feature of carrying cationic moieties in starch macromolecules, revealed rheological properties closest to acetylated starch.

### 3.2. Low Field Nuclear Magnetic Resonance Study 

LF NMR enables studies of the differences in molecular mobility between various food components. They are reflected in the longitudinal (*T*_1_) and transverse (*T*_2_) relaxation times of protons, more often those of water. The separation of multiple types of water protons is based on the deconvolution of *T*_1_ and *T*_2_ measurements [70]. Spin-lattice (*T*_1_) relaxation time describes the transfer of previously absorbed energy from spin to the surrounding environment, whereas spin-spin (*T*_2_) relocation time describes the transfer of previously absorbed energy from spin to neighbouring spins. The first one is related to the ratio of free to entrapped water and second to water molecules dynamics [71]. Entrapped water, however, cannot be identified with bound water, as it is immobilized in foods hydrocolloids as well as in capillaries or cells. If it is released during cutting or damage and can flow freely. Its other properties are the same as those of free water [72].

As it is presented in Table 2, the dissolution of starch in water resulted in a reduction in spin-lattice correlation time values. This means that part of the water has been entrapped in the network of hydrocolloid macromolecules. In pastes prepared with distilled water (no pH regulation), for the majority of tested starches, the changes were between 0.12 to 0.16 s. Only oxidised and OSA starches showed different behaviours. The former was found to form a weaker network in demineralized water while the latter formed a stronger network in this environment. In a buffered environment, the behaviour of these starches changed. While in the case of oxidised starch that contains anionic carboxyl groups the amount of entrapped water was increased, in the OSA starch which contains additional large hydrophobic substituents it was decreased. A slight effect of the presence of buffers on the amount of entrapped water was observed for native starch, distarch phosphate and cationic starch. Nevertheless, a decrease in the difference of *T*_1_ values in pastes and in solvent with an increase in pH can be noticed. This can be explained by the influence of negative phosphate ions contained in the structure of all these starches. Acetylated starch, containing slightly hydrophobic acetyl groups, showed increased ability to entrap water in buffered environment that was independent of pH.

The presence of starch macromolecules dissolved in water also reduced the spin-spin relaxation time *T*_2_ (Table 3). The largest differences were observed in the case of native starch. All modified starches caused smaller *T*_2_ changes. Oxidised starch stands out with *T*_2_ being nearly identical at acidic (2.5) and alkaline (9.5) pH of the environment.

The experimental data of spin-lattice and spin-spin correlation times were used for the calculation of mean correlation time–the parameter describing rotational motions of the water molecules (Table 4). Pure water is characterised by a mean correlation time of 10^−12^ s, whereas for ice this value is 10^−6^ s [73]. In systems with a low concentration of macromolecules, the dipole interactions of the spins are generally averaged. Thus, the molecular dynamics of protons is described by the mean correlation time, defined as the time it takes for the magnetic dipoles of the lattice to rotate by one radian [64]. When analysing the data presented in Table 4, it can be noticed that both in the acidic (pH = 2.5) and alkaline medium, a decrease in the mean correlation time was observed. In neutral buffer (pH = 7.0) and in demineralised water, an increase in *τ_c_* was observed. The only exception was OSA starch, which was found to strongly immobilize water molecules under the conditions of low ionic strength. The water dynamics were most significantly reduced by native starch, regardless of the dispersion medium.

### 3.3. Dynamic Light Scattering and ζ Potential Study 

ζ potential values recorded in the analysed starch samples are presented in Table 5. In all the analysed samples (except for distarch phosphate in water), the starch was negatively charged. In water (non pH regulated solutions) the highest ζ potential value was observed in native starch (−10.02 mV). Modification of starch resulted in reduction in the values (from −8.81 mV for OSA starch to −1.44 mV for cationic starch). During the measurements in buffers, as expected, an increase in the ζ potential value (negative charge) was observed with an increase in the pH of environment. Only in the case of OSA starch the potential at pH 7.0 and 9.5 was at a similar level (−4.63 mV and −4.52 mV, respectively). In solutions with regulated pH, the highest value of ζ potential was recorded for oxidised starch (from −3.99 mV at pH 2.5 to −8.17 mV at pH 9.5).

The z-average value is the intensity weighted mean hydrodynamic diameter of the molecules measured with the DLS method. Z-average results are presented in the Table 6. In turn, the polydispersity index (PdI) is presented in Table 7. This value indicates whether there are mono- or poly-disperse samples in the tested material. The highest PdI value was recorded in the case of native starch (0.840–1.000), OSA starch (0.798–1.000) and distarch phosphate (0.772–0.993). Such high PdI was proved by a very broad hydrodynamic diameter distribution. The lowest values were recorded in the case of cationic starch (0.660–1.000) and acetylated starch (0.560–1.000), although they still indicate high polydispersity of the samples. The smallest PdI was observed in the case of oxidised starch (0.379–1.000). The calculated values of PdI generally show high polydispersity of the tested samples. The range of particle size distribution (hydrodynamic diameter) very wide. For this reason, to better illustrate the changes observed, the size of molecules in the solutions are presented as z-average values. In water (non pH regulated solutions) the highest hydrodynamic diameter was observed for native starch (594.8 nm). Other samples (OSA, distarch phosphate, cationic starch and acetylated starch) showed a clearly smaller z-average (340.6 nm, 258.0 nm, 256.3 nm and 233.2 nm, respectively). Native starch in buffers with pH 2.5–9.5 was characterised by smaller diameters compared to aqueous solution (334.6 nm–307.6 nm, respectively). On the other hand, in the case of acetylated starch, cationic starch and OSA, no significant differences between pH unregulated solutions and buffered solutions were observed. There was also no noticeable relationship between the pH and the hydrodynamic diameter of starch. In the distarch phosphate sample, a significant z-average increase in buffers (480.1 nm–609.5 nm) compared to the non pH regulated solution (258.0 nm) was found. Oxidised starch deserves a separate discussion. The hydrodynamic diameter observed in water (31.1 nm) and buffers with pH in the range 2.5–7.0 (38.1 nm and 33.5 nm, respectively) is clearly lower than all other results. In this case, low z-average values were also accompanied by relatively low polydispersity (0.605; 0.457 and 0.379 in water, buffer at pH 2.5 and 7.0, respectively). However, at pH 9.5, a strong increase in the hydrodynamic diameter to the value 508.7 nm was recorded.

## 4. Discussion

**Figure 1:**
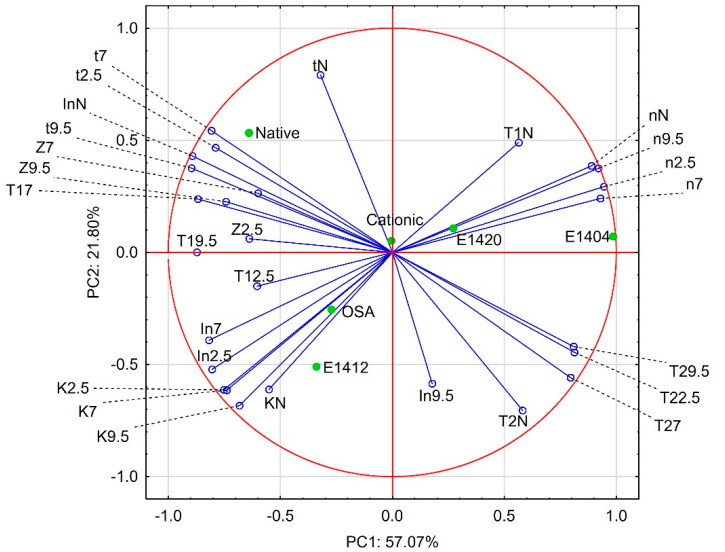
Principal Components Analysis of the data presented in the study; Explanatory notes: K2.5; K7; K9.5; KN–consistency indexes at pH 2.5; 7.0; 9.5 and without pH regulation, respectively; n2.5; n7; n9.5; nN flow behaviour indexes at pH 2.5; 7.0; 9.5 and without pH regulation, respectively; T12.5; T17; T19.5; T1N–spin-lattice relaxation times at pH 2.5; 7.0; 9.5 and without pH regulation, respectively; T22.5; T27; T29.5; T2N–spin-spin relaxation times at pH 2.5; 7.0; 9.5 and without pH regulation, respectively; t2.5; t7; t9.5; tN–mean correlation times at pH 2.5; 7.0; 9.5 and without pH regulation, respectively; Z2.5; Z7; Z9.5–ζ potential values at pH 2.5; 7.0; and 9.5, respectively; In2.5; In7; In9.5; InN–intensity weighted mean hydrodynamic diameter at pH 2.5; 7.0; 9.5 and without pH regulation, respectively.

All of the methods applied in our study enable studies on starch-solvent interaction. This problem has been described most broadly in relation to rheological studies [11,12,13,14,15,16,17,18,19,20,21,22,23,24,25,26,27]. Nevertheless, the other two methods used seem to be designed to study these interactions [34,35,36,37,74]. Moreover, a clear relationship between rheological and LF NMR studies has been proven [75]. In the case of electrokinetic studies, the effect of environment on the obtained results manifests itself in the necessity to use solutions with an appropriate ionic strength for analyses [45,46]. In order to verify whether there is any correlation between the results obtained with the three methods used, and to determine to what extent these methods are mutually complementary or substitutary, the Principal Component Analysis was carried out and its results are presented in Figure 1. The results of the ζ potential measurements in the distilled water environment were not taken into account in this analysis for the reasons presented above. The two principal components (factor 1 and 2) explain 78.87% of total variance, therefore, the conclusions on the basis of this PCA analysis can be considered legitimate.

As can be seen at Figure 1, the parameters describing rheological properties of starch i.e., consistency indexes (K) and flow behaviour indexes (*n*) formed bunches of lines located close to each other on the loadings plot. Their mutual localization confirmed a common knowledge that a decrease in consistency index is accompanied by increase in convergence with the Newtonian flow of starch pastes [33]. Moreover, data of spin-spin correlation times (*T*_2_) as well as of ζ potential (Z) also formed bunches of lines located close to each other on the loadings plot. This indicates that the values of these parameters are primarily influenced by the properties of the starches themselves and, to a lesser extent, by environmental conditions. In contrast, position of lines describing spin-lattice relaxation time (*T*_1_) as well as intensity weighted mean hydrodynamic diameter indicate a strong influence of the environment on these parameters. This suggests that these parameters are more sensitive to the environmental conditions in which starch macromolecules are dispersed.

The position of the bunches of lines on the biplot (Figure 1) also shows the relationship between the analysed parameters. The strongest correlation can be observed between ζ potential (Z) and LF NMR parameters i.e., spin-spin relaxation times (*T*_2_) and mean correlation time (t). This suggest that ζ potential of dispersed macromolecules determines dynamics of water molecules constituting the continuous phase. This relationship has not been reported in the literature so far.

The relationship between rheological properties and LF NMR parameters [64,75] has been described in the literature. However, the relations observed in this study are not obvious and simple. Primarily, spin-lattice relaxation time values, that are related to the ratio of free to entrapped water, are expected to be in strong correlation with rheological parameters [64,71,75]. However, the dependence of spin-lattice relaxation time on environmental conditions is much stronger than that of rheological parameters (Figure 1) Despite this, the latter shows a strong dependence on the presence of low molecular mass solutes (Table 1).

**Figure 2:**
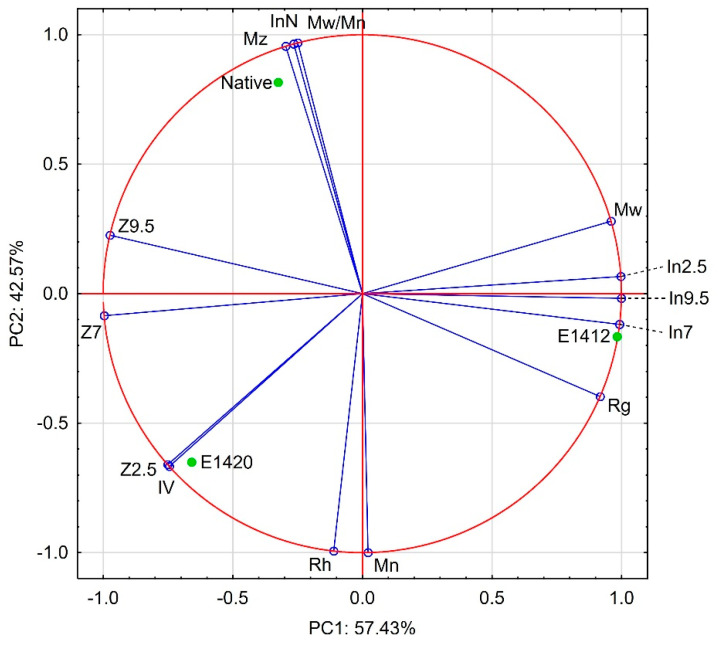
Principal Components Analysis of the data presented in Table 6 and Table 7, as well as derived from [76]; Explanatory notes: Z2.5; Z7; Z9.5—ζ potential values at pH 2.5; 7.0; and 9.5, respectively; In2.5; In7; In9.5; InN—intensity weighted mean hydrodynamic diameter at pH 2.5; 7.0; 9.5 and without pH regulation, respectively; M_n_—number average molecular mass; M_w_—weight average molecular mass; M_z_—z-average molecular mass; Mw/Mn—polydispersity; IV—intrinsic viscosity; R_g_—radius of gyration; R_h_—hydrodynamic radius.

All investigated preparations were scattered throughout the whole area of the biplot (Figure 1). The most distant was native starch, containing no modifying groups and oxidised starch-distinguished by a much lower molecular weight than the other preparations. The molecular mass seems to be the most decisive factor for PC2 as the position of modified starches, the cationic and acetylated starches as well as sodium starch octenylsuccinate, on the score plot was close to the center. The results of PCA analysis of the data obtained by electrokinetic and DLS studies as well as from SEC analyses of three starches: native, acetylated and distarch phosphate are presented in Figure 2.

Particular attention should be paid to understanding the data obtained as a result of electrokinetic and dynamic light scattering studies. With regard to starch, these studies have so far only been carried out for the dispersion of starch in granular form or its solid nanoparticles [48,49,50,51,52,53,54,55,56]. It is all the more interesting as the phenomenon of light scattering is also used to determine the molecular weight of polymers. Both methods, dynamic and static light scattering, have their limitations and allow for the analysis of the status of macromolecules in dispersion. Moreover, some of the parameters derived from both analyses, such as radius of gyration R_g_ and hydrodynamic radius R_h_, seem to be the same [14,45,46,47]. However, the appropriate measurement of molecular mass distribution by SEC analysis followed by static light scattering requires molecular distribution of polymer in solution, while the DLS method was designed for studies of dispersion of solid particles [14,45,46,47,77,78]. Relevant data on the molecular mass distribution obtained with SEC for, among others, three food grade starches of the same degree of substitution was recently published [76] and was used in the analysis. The data used for PCA analysis was included as supplementary data Table S1. The results proved excellent coverage of the data, as the two principal components (factor 1 and 2) explain 100% of total variance. However, since the PCA analysis was carried out on the basis of the analytical data for three starch samples only, the observed relationships cannot be considered as indisputable. Comparing the data derived from different analytical methods, primarily a strong correlation between ζ potential value at pH = 2.5 and intrinsic viscosity could be observed. ζ potential values at pH = 7.0 and pH = 9.5 correlated with radius of gyration and weight average molecular mass, respectively. Despite such a perfect correlation, it cannot be said that the observed relationship between these parameters is a rule. Further studies are necessary to conform or exclude the above hypothesis.

The relationship between z-average molecular mass and intensity weighted mean hydrodynamic diameter seems to be very interesting, as both parameters were determined based on light scattering phenomena. As presented in Figure 2, there is a strong relationship between M_z_ and intensity weighted mean hydrodynamic diameter, but only in the case of the unbuffered paste. Data measured for pastes containing buffers revealed only minor correlation between M_w_ and R_g_. The presence of low molecular mass solutes in these pastes significantly affects the conformation of starch macromolecules and, in consequence, their dimensions. Analysis of unbuffered paste most closely reproduces the conditions of SEC analysis which results in such a good correlation.

## 5. Conclusions

All of the methods applied in our study enable investigation of both the properties of starches and their interaction with the dispersing environment.

- Rheological studies showed that the pH buffering substantially decreased values of consistency indexes. The pH value itself was found not significant. Moreover, the decrease in consistency index is accompanied by increase in convergence with the Newtonian flow of starch pastes, so that in buffered pastes almost all starches revealed Newtonian-like behaviour. Cross-linking is the only modification that resulted in the increase in the consistency index; slight changes were observed for OSA, more significant for cationic and acetylated starches. Oxidised starch, manifesting the most pronounced changes, stand out from the other starches tested;
- LF NMR studies indicated that dissolution of starch in water resulted in a reduction in spin-lattice as well as spin-spin relaxation times. Environmental conditions had a stronger effect on spin-lattice relaxation time, whereas spin-spin correlation time was primarily influenced by the properties of the starches themselves. Moreover, the changes in relaxation times followed the patterns observed in rheological studies;
- Electrokinetic and DLS studies showed that potential values are primarily influenced by the properties of the starches themselves and, to a lesser extent, by the environmental conditions. In contrast, intensity weighted mean hydrodynamic diameter values were more sensitive to the environmental conditions in which starch macromolecules are dispersed.

The conducted research also showed complementarity and, to some extent, substitutability of the applied research methods and size exclusion chromatography (a method not used in this work). ζ potential of the dispersed macromolecules is determined by dynamics of water molecules constituting the continuous phase. It is also reflected by the spin-spin relaxation time. Spin-lattice relaxation time, as well as intensity weighted mean hydrodynamic diameter, were more sensitive to the environmental conditions of the system within which starch macromolecules were dispersed. Moreover, there is a strong relationship between M_z_ and intensity weighted mean hydrodynamic diameter but only if no other solutes are present in the paste.

## Figures and Tables

**Table 1:** Parameters of Ostwald-de Waele rheological equation for the analysed starch samples.

		Native Starch	Acetylated Starch	Cationic Starch	OSA Starch	Distarch Phosphate	Oxidised Starch
No pH regulation	K	0.092 ± 0.002	0.007 ± 0.000	0.015 ± 0.000	0.080 ± 0.003	0.302 ± 0.007	0.001 ± 0.000
	n	0.661 ± 0.004	0.906 ± 0.003	0.819 ± 0.000	0.711 ± 0.006	0.477 ± 0.000	1.102 ± 0.001
	r	0.9998	1	1	0.9995	0.9999	0.9988
pH = 2.5	K	0.013 ± 0.000	0.004 ± 0.000	0.009 ± 0.000	0.022 ± 0.000	0.025 ± 0.001	0.001 ± 0.000
	n	0.824 ± 0.001	0.941 ± 0.003	0.869 ± 0.000	0.793 ± 0.009	0.777 ± 0.002	1.130 ± 0.007
	r	1	1	1	0.9998	1	0.9985
pH = 7.0	K	0.013 ± 0.000	0.004 ± 0.000	0.009 ± 0.000	0.024 ± 0.001	0.025 ± 0.000	0.001 ± 0.000
	n	0.827 ± 0.002	0.945 ± 0.001	0.869 ± 0.001	0.791 ± 0.003	0.774 ± 0.000	1.310 ± 0.003
	r	1	1	1	0.9998	0.9999	0.9984
pH = 9.5	K	0.014 ± 0.000	0.004 ± 0.000	0.009 ± 0.000	0.026 ± 0.001	0.035 ± 0.001	0.001 ± 0.000
	n	0.821 ± 0.001	0.945 ± 0.003	0.866 ± 0.003	0.797 ± 0.005	0.719 ± 0.001	1.133 ± 0.007
	r	1		1	0.9997	0.9998	0.9984

**Table 2:** Spin-lattice relaxation time *T*_1_ values [s] for the analysed starch samples.

	Solvent	Native Starch	Acetylated Starch	Cationic Starch	OSA Starch	Distarch Phosphate	Oxidised Starch
No pH regulation	2.44	2.32	2.30	2.29	2.18	2.28	2.38
pH = 2.5	2.39	2.25	2.15	2.27	2.29	2.23	2.19
pH = 7.0	2.40	2.30	2.19	2.27	2.23	2.25	2.17
pH = 9.5	2.36	2.32	2.14	2.27	2.32	2.25	2.13

**Table 3:** Spin-spin relaxation time *T*_2_ values [s] for the analysed starch samples.

	Solvent	Native Starch	Acetylated Starch	Cationic Starch	OSA Starch	Distarch Phosphate	Oxidised Starch
No pH regulation	1.95	1.14	1.64	1.49	1.63	1.57	1.61
pH = 2.5	2.05	1.06	1.84	1.83	1.52	1.72	1.99
pH = 7.0	1.80	1.03	1.46	1.43	1.38	1.42	1.57
pH = 9.5	1.99	1.32	1.56	1.76	1.59	1.69	1.97

**Table 4:** Mean correlation time values *τ_c_* [10^−8^ s] for the analysed starch samples.

	Native Starch	Acetylated Starch	Cationic Starch	OSA Starch	Distarch Phosphate	Oxidised Starch
No pH regulation	33.5	27.7	28.9	26.3	28.0	28.9
pH = 2.5	33.7	24.4	25.8	28.6	26.2	23.9
pH = 7.0	34.9	27.9	29.2	29.2	29.1	26.5
pH = 9.5	31.1	26.4	26.3	28.3	26.6	23.4

**Table 5:** ζ potential of analyzed starch samples [mV] for the analysed starch samples.

	Native Starch	Acetylated Starch	Cationic Starch	OSA Starch	Distarch Phosphate	Oxidised Starch
No pH regulation	−10.02 ± 1.02	−2.88 ± 0.32	−1.44 ± 0.62	−8.81 ± 1.76	2.86 ± 1.20	−6.39 ± 1.42
pH = 2.5	−1.50 ± 0.19	−0.78 ± 0.06	−1.24 ± 0.13	−2.08 ± 0.37	−1.62 ± 0.15	−3.99 ± 0.89
pH = 7.0	−2.05 ± 0.23	−1.65 ± 0.52	−2.05 ± 0.46	−4.63 ± 1.01	−3.06 ± 0.44	−6.56 ± 0.71
pH = 9.5	−2.99 ± 0.40	−3.05 ± 0.62	−3.32 ± 0.18	−4.52 ± 0.93	−4.54 ± 0.98	−8.17 ± 1.34

**Table 6:** Intensity weighted mean hydrodynamic diameter (z-average) recorded in the analysed starch samples [nm].

	Native Starch	Acetylated Starch	Cationic Starch	OSA Starch	Distarch Phosphate	Oxidised Starch
No pH regulation	594.8 ± 98.1	233.2 ± 11.5	256.3 ± 7.9	340.6 ± 21.7	258.0 ± 100.3	31.1 ± 10.4
pH = 2.5	334.6 ± 47.8	245.6 ± 5.2	301.2 ± 34.7	351.9 ± 38.9	579.6 ± 35.0	38.1 ± 0.3
pH = 7.0	322.7 ± 33.2	308.4 ± 28.0	293.6 ± 2.3	307.1 ± 27.3	480.1 ± 69.0	33.5 ± 0.5
pH = 9.5	307.6 ± 1.0	238.8 ± 1.2	295.0 ± 28.9	319.0 ± 17.3	609.5 ± 48.1	508.7 ± 10.2

**Table 7:** Mean polydispersity index (PdI) recorded in the analysed starch samples.

	Native Starch	Acetylated Starch	Cationic Starch	OSA Starch	Distarch Phosphate	Oxidised Starch
No pH regulation	0.949	1.000	1.000	1.000	0.772	0.605
pH = 2.5	0.840	0.600	0.834	0.798	0.947	0.457
pH = 7.0	0.884	0.735	0.660	0.959	0.848	0.379
pH = 9.5	1.000	0.560	0.812	0.965	0.993	1.000

## Data Availability

All data generated or analyzed during this study are included in this published article (and its supplementary information files).

## Supplementary Materials

The following supporting information can be downloaded at: https://www.mdpi.com/article/10.3390/polym14152977/s1, Table S1: SEC analysis results of modified starch preparations used for PCA analysis presented in Figure 2.
