# Supplementary material for: Polymer-Solvent Interactions in Modified Starches Pastes–Electrokinetic, Dynamic Light Scattering, Rheological and Low Field Nuclear Magnetic Resonance Approach
Source: Polymers (Basel). 2022 Jul 22;14(15):2977. doi: 10.3390/polym14152977 (PMC9331432; doi:10.3390/polym14152977)
Supplement: Supplementary file 1 [file polymers-14-02977-s001.zip › polymers-1816717-supplementary.pdf]

Table S1. SEC analysis results of modified starch preparations used for PCA analysis presented in Figure 2.

Starch	M_n	M_w	M_z	M_w/M_n	IV	R_h	R_g	R_g/R_h	M-H a	Branches
Native	2520000	28700000	610000000	11.52	1.407	79	96	1.22	0.279	1010
E 1412	20900000	26400000	32200000	2.46	1.130	85	187	2.19	0.459	2481
E1420	15200000	31400000	51500000	1.09	1.790	90	126	1.40	0.420	1250

Source: *Processes* **2022**, *10*, 938. https://doi.org/10.3390/pr10050938
